# Optimising Time-Frequency Distributions: A Surface Metrology Approach

**DOI:** 10.3390/s23135804

**Published:** 2023-06-21

**Authors:** Damir Malnar, Miroslav Vrankic

**Affiliations:** Faculty of Engineering, University of Rijeka, Vukovarska 58, 51000 Rijeka, Croatia; mvrankic@riteh.hr

**Keywords:** optimisation, time-frequency, distribution, surface, metrology, evolutionary, multiform, kernel, Abbot, curve

## Abstract

Time-frequency signal processing offers a significant advantage over temporal or frequency-only methods, but representations require optimisation for a given signal. Standard practice includes choosing the appropriate time-frequency distribution and fine-tuning its parameters, usually via visual inspection and various measures—the most commonly used ones are based on the Rényi entropies or energy concentration by Stanković. However, a discrepancy between the observed representation quality and reported numerical value may arise when the filter kernel has greater adaptability. Herein, a performance measure derived from the Abbot–Firestone curve similar to the volume parameters in surface metrology is proposed as the objective function to be minimised by the proposed minimalistic differential evolution variant that is parameter-free and uses a population of five members. Tests were conducted on two synthetic signals of different frequency modulations and one real-life signal. The multiform tiltable exponential kernel was optimised according to the Rényi entropy, Stanković’s energy concentration and the proposed measure. The resulting distributions were mutually evaluated using the same measures and visual inspection. The optimiser demonstrated a reliable convergence for all considered measures and signals, while the proposed measure showed consistent alignment of reported numerical values and visual assessments.

## 1. Introduction

Real-life signals, whether natural or artificial, frequently require joint time-frequency analysis to better understand the internal signal structure due to such signals’ amplitude and frequency temporal variability. Time-frequency representations result from various time-frequency distributions and are methods of describing temporal variations of signals’ spectral content [1]. In general, there are linear, quadratic and higher-order classes of distributions with applications investigated in diverse fields of research, such as instantaneous frequency estimation [2,3,4,5,6], parameter determination [7,8,9], signal modelling [10,11,12], wireless resource management [13,14], target localisation [15,16,17], and biomedical signal abnormalities [18,19,20,21,22,23]. Time-frequency representations augment and extend the classical analysis by offering additional features that can help better discriminate and classify diverse phenomena, and this is paramount for emerging pattern recognition and machine learning applications [24]. However, the fundamental challenge in successfully applying time-frequency analysis and processing methods lies in the appropriate choice of the distribution and its filtering kernel parameters.

According to the literature, the quadratic class of distributions is of the highest interest since it has a signal composition displayed as distinct concentrations of energy along the time-frequency (t,f) plane that resemble a landscape of ridges of varying heights. Crests of these ridges correspond to signal components’ instantaneous frequency (IF) and amplitude laws [1]. From the practitioner’s perspective, the most valuable quadratic time-frequency distributions (QTFDs) are often found to be the spectrogram, smoothed pseudo-Wigner–Ville (SPWVD), extended modified B (EMBD) and compact kernel distribution (CKD) [1,25]. Although quadratic in the second step similar to the squared magnitude of the short-time Fourier transform (STFT), the spectrogram has become common practice in (t,f) analysis because of its strong relationship to classical signal processing and the few required parameters. Nonetheless, experts regularly criticise the spectrogram for the trade-off between the component time and frequency resolution tied to the width of the analysis window, which motivates the study and deployment of other potentially superior quadratic time-frequency distributions. However, as illustrated by the Wigner–Ville distribution (WVD), the quadratic character of the transform not only builds and enhances the concentration of signal components as auto-terms but also generates their cross-terms. Cross-terms often create deceptive patterns and mask natural features when there is non-linear frequency modulation or more than one constituent component in the signal [1,26]. They are commonly seen as interferences and a considerable impediment to the proper interpretation and use of the QTFDs. However, they may be helpful as extra classification features, or their geometry could be exploited as in [27]. In contrast to the spectrogram, cross-terms in the WVD do not vanish with distance, but appear precisely at the midpoint and in an oscillatory pattern proportional to the (t,f) separation of interacting signal parts. Therefore, the compromise in the quadratic class becomes the ratio of auto-terms to cross-terms reflected in distributions’ kernel parameters.

More parameters allow additional freedoms in kernel topology adaptation to pass signal auto-terms while suppressing cross-terms. Ultimately, the result should be a visually accurate, mostly sparse time-frequency distribution (TFD) with diminished interferences and signal energy highly concentrated under the components’ IF laws. In order to accomplish higher performance, TFDs often depart from the sound mathematical properties of the WVD in favour of representation clarity and informativeness [1]. Besides greater kernel adaptability that can result in a better-performing TFD, more parameters add to the complexity of design and optimisation. Choosing the appropriate values in the case of an unknown signal is not a simple task even for a spectrogram using a single-parameter analysis window, and possible parameter interdependencies additionally compound the problem. The energy distribution across the (t,f) plane follows arbitrary directions in the general signal. However, independent filtering parallel to domain axes used in separable kernel distributions such as CKD, EMBD and SPWVD often yields satisfactory results for various signals in practice. Nevertheless, directional filtering can still perform better, as evident from directional CKD [1,28] or adaptive directional time-frequency distribution [29]. Although similar approaches were suggested previously, for instance, multiform tiltable exponential kernel (MTEK) [30] or radially Gaussian kernel (RGK) [31], the sheer number of parameters and dynamic optimisation requiring additional user-supplied meta-parameters hinder their practical application. Regardless, optimisation with an appropriate performance assessment is crucial and is still an open issue.

Because the selection of kernel parameters based on visual judgement is commonly criticised for being subjective, researchers proposed various methodologies to objectively evaluate TFD performance in terms of desirable attributes for high-performing distributions [1]. Jones and Parks proposed a concentration measure based on the ratio of higher distribution norms as a performance indicator. However, the amplitude sensitivity of the measure made the resulting kernel emphasise high-power components, so an additional (t,f) region selecting parameter that must be supplied was introduced [32]. Nevertheless, measures derived from lower-order norms, such as Stanković’s energy concentration measure (ECM), proved more robust [33]. Initially used for S-method optimisation, this measure was also found to be appropriate for the performance evaluation of different TFDs [34]. Commonly found in signal sparsity estimation, the lower order norms $\ell_{0}$, $\ell_{1}$, $\ell_{2}$ norm and Gini index were also investigated as performance indicators in [35]. Albeit not directly suitable as a performance measure due to its noise sensitivity, a threshold variant of the $\ell_{0}$ norm was successfully applied in automatic SPWVD optimisation in [36].

Besides the concentration, TFD performance is commonly measured as the distribution complexity using generalised Rényi entropy with different normalisations applied in practice [1,37]. In [38], Sucic et al. based their instantaneous number of signal components estimation algorithm on the short-term Rényi entropy. Saulig et al. later used it in spectrogram optimisation [39]. To define performance differences and selection criteria between various TFDs, Boashash and Sucic introduced the normalised instantaneous resolution (NIR) measure based on per-time slice measurements of classical spectral performance indicators [40]: components’ instantaneous bandwidths, side-lobe to main-lobe and cross-term to main-lobe amplitude ratios of two spectrally closest peaks. Although in-depth, the method proved too complicated for TFD optimisation due to its closest peak-finding sensitivity and because it requires an already well-performing exploratory distribution to identify the cross-terms [25]. The Reinhold measure, which was subsequently proposed, attempts to overcome NIR issues but introduces two additional user-supplied parameters while limiting the analysis to only two components at any moment [41].

The optimality of a time-frequency distribution is usually defined relative to the ideal representation, whereas the WVD has been found to only be optimal for a mono-component linear frequency modulated (LFM) signal [1]. Exploiting this optimality, Al-Sa’d et al. [25] proposed decomposing the signal into LFM segments from which a piece-wise spline WVD is constructed and used as a reference for comparing the accuracy and resolution of other TFDs. Based on the proposed performance evaluation process, popular distributions such as CKD, SPWVD and EMBD have again been recommended for the general case of multi-component non-stationary and non-linear instantaneous frequency laws signals.

Rényi entropy and Stanković’s ECM are among the more commonly used measures [24,25]. As a global performance indicator in the form of a single value, such measures are convenient for implementing TFD optimisation algorithms. When stated as the general optimisation problem with only the analysed signal and TFD kernel parameters as input arguments, such algorithms allow simple modular inclusion into machine learning frameworks. However, a quantitatively optimised filtering kernel may distort some or all of the information in the resulting TFD, making a visual evaluation by a signal specialist indispensable. In addition to numerically capturing TFD performance, a credible metric should also align with how the TFD is visually perceived to perform.

In this study, our objective was to define a global measure relative to the uniform time-frequency distribution and agreeable with the visual assessment using definitions from the surface metrology as inspiration for the TFD performance indicators.

A product’s texture and surface properties are often critical to functional specifications, and material sciences and manufacturing have long recognised the need for controlling and appropriately measuring those properties. Many parameters to capture and quantify surface complexities of processed materials were previously used in national standards, which resulted in what was known as the “parameter rash” [42]. In order to address such a situation, the regulating bodies under the SURFStand project [43] put forward a set of parameters based on statistical, functional and morphological surface features that ultimately became accepted and are continuously developed as the ISO-25178 international standard for geometric product specification [44]. Of particular interest, we found definitions of aerial surface texture parameters that served as the basis for the proposed measure.

## 2. Materials and Methods

Essentially, a quadratic (t,f) distribution, denoted $\rho_{z}\left(t,f\right)$, is a height map and can be viewed as a surface texture that results from (t,f) smoothing of the rough WVD in the process defined as [1]:

(1) $$\rho_{z}\left(t,f\right)=\gamma \left(t,f\right)\underset{\left(t,f\right)}{**}W_{z}\left(t,f\right),$$

where γ(t,f) is the filter kernel convolved (∗∗) with the Wigner–Ville distribution $W_{z}\left(t,f\right)$ of the complex signal z(t). The WVD, in turn, results from the Fourier transform (FT) over the time-lag variable τ of the signal instantaneous auto-correlation function (IAF), denoted $K_{z}\left(t,\tau \right)$, as in Equation (2):

(2) $$W_{z}\left(t,f\right)=\underset{\tau \to f}{F}\{K_{z}\left(t,\tau \right)\}=\underset{\tau \to f}{F}\left\{z\left(t+\frac{\tau }{2}\right)z^{*}\left(t-\frac{\tau }{2}\right)\right\},$$

where F is the forward FT, and z(t) is the analytic associate of the real signal s(t).

However, QTFDs are usually designed in the Doppler-lag (ν,τ) domain and then transformed back to the (t,f) domain as in Equation (3):

(3) $$\rho_{z}\left(t,f\right)=\underset{\tau \to f}{F}\left\{\underset{t\leftarrow \nu }{F^{-1}}\left\{g\left(\nu ,\tau \right)A_{z}\left(\nu ,\tau \right)\right\}\right\},$$

where $F^{-1}$ is the inverse FT, g(ν,τ) is the kernel in the Doppler-lag form and $A_{z}\left(\nu ,\tau \right)$ is the signal ambiguity function (AF) constructed from the IAF, or, equivalently, from the WVD as in Equation (4) [1]:

(4) $$A_{z}\left(\nu ,\tau \right)=\underset{t\to \nu }{F}\left\{K_{z}\left(t,\tau \right)\right\}=\underset{t\to \nu }{F}\left\{\underset{\tau \leftarrow f}{F^{-1}}\left\{W_{z}\left(t,f\right)\right\}\right\}.$$

Aside from efficient filtering, the Doppler-lag domain makes kernel design easier since it provides some separation between the cross-terms and auto-terms. Filter kernels in the (ν,τ) form are commonly the low-pass type that preserves the signal AF around its origin and de-emphasises locations further away, usually occupied by cross-terms. No smoothing is performed if the kernel is the all-pass type, i.e., g(ν,τ)=1, and the result is the WVD. Conversely, if the kernel is zero everywhere but the signal AF origin, it will flatten the resulting TFD by distributing the signal energy uniformly across the (t,f) plane. Compared to the uniform, any other TFD of the same signal is more informative and can be considered better-performing, so we use it as the reference for the proposed metrics in this study.

In order to establish the metrics, we follow the approach from the surface metrology by first constructing the Abbot–Firestone curve of a discrete TFD. The Abbot curve is more frequently known as the bearing area or material ratio curve (MRC) because of its usage in definitions of functional surface parameters [45]. Essentially, the Abbot curve is a form of an empirical cumulative function that shows the ratio of a surface cross-sectional area at a considered height to the evaluation area.

The material ratio curve of a discrete (t,f) distribution $\rho_{z}\left[n,k\right]$ of size N·M, with *n* and *k* being discrete-time and frequency index, respectively, can be formed as the finite sequence of non-increasing values:

(5) $$\left(h_{1},h_{2},\cdots ,h_{N\cdot M}\right),\;h_{i}\geq h_{i+1},$$

obtained from $\rho_{z}\left[n,k\right]$ as the centred samples [45]

(6) $$h_{i}=\rho_{z}\left[n,k\right]-m_{\left[n,k\right]},\;i\in \left\{1,\cdots ,N\cdot M:i\in N\right\},$$

where $m_{\left[n,k\right]}$ is the average of $\rho_{z}\left[n,k\right]$ defined as [1]

(7) $$m_{\left[n,k\right]}=\frac{1}{NM}\sum_{n=0}^{N-1}\sum_{k=0}^{M-1}\rho_{z}\left[n,k\right].$$

**Figure 1:**
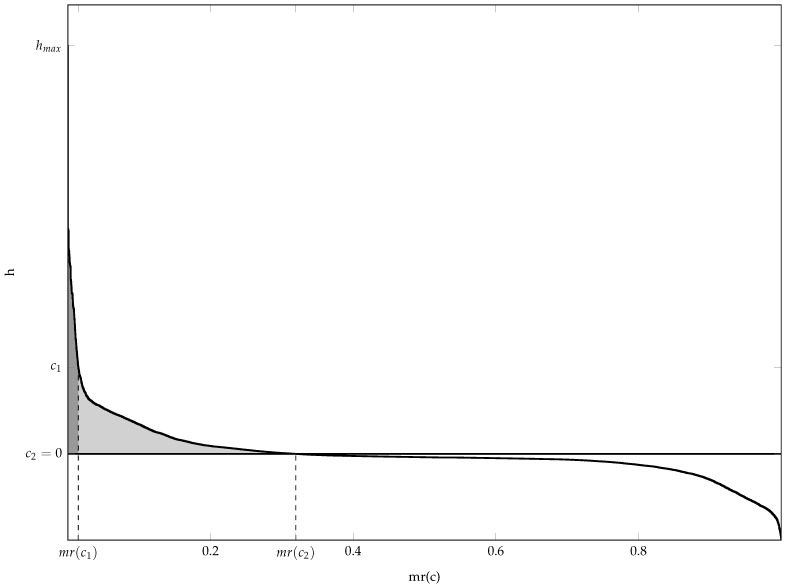
The material ratio curves involved in the definitions of the proposed performance indicators. The horizontal axis is the areal material ratio mr, and the vertical axis is the height *h* of the surface cross-section at the considered material ratio relative to the reference surface, i.e., the uniform TFD. The curves of the uniform (flat line at $c_{2}=0$) and Wigner–Ville distribution (S-shaped curve) of the example signal defined in Equation (24) consisting of two crossed LFM components as per Equations (25) and (), respectively, are shown. The shaded area is the material volume $Vm(mr\left(c_{2}\right))$, while the smaller, darker part is the material volume $Vm(mr\left(c_{1}\right))$ corresponding to the significant auto-terms.

Figure 1 shows the resulting MRCs of the uniform and the WVD of an example signal consisting of two crossed LFM components. The horizontal axis represents the areal material ratio mr, while the vertical axis is the height *h* of the surface cross-section at the considered material ratio relative to the reference surface, which is the uniform TFD.

For a considered level *c* and the evaluation area equalling the size of $\rho_{z}\left[n,k\right]$, we calculate mr(c) as in Equation (8):

(8) $$mr\left(c\right)=\frac{1}{NM}\sum_{i}{\left(h_{i}\right)}^{0},\;i\in \left\{1,\cdots ,N\cdot M:h_{i}\geq c\right\},$$

which is essentially a threshold form of the $\ell_{0}$ norm. The area between an MRC and the intersecting line at *c* or at mr(c) represents a volume. For a given mr, similar to the volume parameters from the surface metrology [45], we define the area under the positive section of an MRC as the material volume Vm(mr), which corresponds to positive energy in $\rho_{z}\left[n,k\right]$. As $\rho_{z}\left[n,k\right]$ can be considered a densely sampled surface, a straightforward summation can be used to calculate the volume Vm(mr) as in Equation (9):

(9) $$Vm\left(mr\right)=\sum_{i}h_{i},\;i\in \left\{1,\cdots ,N\cdot M:h_{i}\geq h\left(mr\right)\wedge h\left(mr\right)>0\right\}.$$

As is evident in Figure 1, Vm(·) of the WVD at mr(0) has an equal-area counterpart between level zero and the negative of the MRC since $h_{i}$ samples are centred. The shape of this area and its deepest recess can be used to estimate the cross-term contribution to the material volume due to their oscillatory nature. By comparing the shapes of the positive and negative areas, it is clear how cross-terms make a substantial part of the material volume of the WVD and increase mr(0). In contrast, the uniform TFD has no material volume, but it takes the entire evaluation area N·M since its MRC is the line at level zero. These observations demonstrate that the MRC of the optimal TFD should be nearly flat in its negative part with the material ratio mr(0) smaller than that of the WVD.

Based on the above insight, the relevant indicators are then the material volume and ratios at levels $c_{1}=\left|\min(h_{i})\right|$ and $c_{2}=0$, respectively, from which the proposed performance metrics follow as

(10) $$p_{1}=mr\left(c_{1}\right),\;0\leq p_{1}\leq 1,$$

(11) $$p_{2}=1-\frac{Vm\left(mr(c_{1})\right)}{V^{+}},\;0<p_{2}\leq 1,$$

(12) $$p_{3}=1-\frac{Vm\left(mr(c_{2})\right)}{V^{+}},\;0<p_{3}\leq 1,$$

with mr(·) and Vm(·) calculated according to Equations (8) and (9), respectively. $V^{+}$ is the positive volume of $\rho_{z}\left[n,k\right]$ calculated as

(13) $$V^{+}=\sum_{n=0}^{N-1}\sum_{k=0}^{M-1}\rho_{z}\left[n,k\right],\;\rho_{z}\left[n,k\right]:\rho_{z}\left[n,k\right]>0.$$

Since $p_{1}$ can be close to zero for nearly flat distributions or the WVD, $p_{3}$ is needed to discriminate higher concentrations further. However, $p_{1}$ and $p_{3}$ can promote cross-terms, so $p_{2}$ provides the counterbalance by only considering values greater or equal to $c_{1}$, i.e., the significant auto-terms, as the principal concentration contributors. For the uniform TFD, $p_{1}$, $p_{2}$ and $p_{3}$ all equal one, so it is uniquely ranked as the worst-performing TFD. On the other extreme, in the ideal TFD case, the *p* values are minimal, and it holds that $p_{2}=p_{3}$.

The so-defined *p* metrics evaluate the performance of $\rho_{z}\left[n,k\right]$ based on its values, but, in general, $\rho_{z}\left[n,k\right]$ may not strictly uphold the energy conservation property and, therefore, may not correspond well with the actual signal. Round-off errors, non-compact support kernels and aliasing in discrete realisations of (t,f) distributions can violate this property. However, most of the signal’s energy should be preserved within the evaluation area of size N·M. Otherwise, it can be argued that a (t,f) distribution no longer represents the signal’s energy distribution. In order to measure the relative amount of this violation, we introduced a discrepancy metric between the average across the whole matrix of $\rho_{z}\left[n,k\right]$ and the average power of the analysed signal z[n] as

(14) $$p_{4}=\left|1-\frac{m_{\left[n,k\right]}}{{}^{1}\;{/}_{N}\sum_{n=0}^{N-1}{\left|z\left[n\right]\right|}^{2}}\right|,\;0\leq p_{4}<1.$$

Smaller *p* values indicate a better-performing TFD, but all *p* metrics must be considered simultaneously and equally important, thus presenting a multi-objective optimisation problem with equally weighted individual objectives. In this sense, we formulated the proposed measure as

(15) $$PM=\frac{1}{4}\sum_{j=1}^{4}p_{j},\;0<PM<1,$$

that is, the average of the proposed *p* metrics. As a single-valued global indicator, the proposed measure PM can directly serve as the objective function in a TFD optimisation algorithm. The problem of selecting optimal kernel parameters in such an algorithm can generally be stated as

(16) $$\underset{kernel\;parameters}{\arg\min}=PM\left(z\left[n\right],\rho_{z}\left[n,k\right]\right),$$

that is, find the kernel parameters for which some performance measure, such as Equation (15), (22) or (23), attains a minimal value globally. Within such a framework, there are three parts to an algorithm then: the kernel, the performance measure, and the core optimiser. As a solution to the posed problem, kernel parameters should be globally optimal; therefore, the core optimiser must also be of the global type. From the plethora of algorithms for global optimisation, we chose the differential evolution (DE) because of its proven effectiveness, algorithmic simplicity and the small number of control parameters.

Differential evolution is an instance of the evolutionary class of global optimisers in evolutionary computing. It is a stochastic direct search, a derivative-free algorithm similar to other popular methods such as the genetic algorithm [46] and particle swarm optimisation [47]. The DE principle of operation is based on the iterative transformation of a population of candidate solutions by subjecting them to rounds of mutation, recombination, assessment and selection. New solutions are produced by perturbing the current population members with their weighted differences calculated between two or more randomly chosen individuals [48,49]. Combined with the selection operation, this has the effect of a self-scaling step when sampling the solution space providing population members with a local contour-matching property that allows fast identification of attractive basins that might contain the global optimum [50].

The surveyed literature shows that there are many DE variants and flavours of those variants [51,52]. Differences between the proposed improvements are commonly used in choosing and adapting the values of the control parameters and what search strategy or combination of strategies are applied and possibly augmented with other search algorithms [52,53]. No variant can be singled out as the best and certainly none are universal. When algorithmic performance is evaluated according to selected benchmark functions commonly used in competitions [52], the majority shows some advantage over the basic algorithm proposed in [49]. However, the original DE with fixed, appropriately set control parameter values is still competitive.

The successful application of DE strongly depends on the control parameters: population size $N_{P}$, difference vector scaling factor *F* and the crossover rate $C_{r}$ [52]. However, if those could be removed while retaining the same search ability, a genuine black-box optimiser could be achieved. Here, we present a minimalistic DE variant as a straightforward black-box search algorithm to provide reliable results when optimising (t,f) distributions. The pseudo-code listing in Algorithm 1 presents the general structure of the proposed optimiser.

**Algorithm 1:** Proposed optimiser

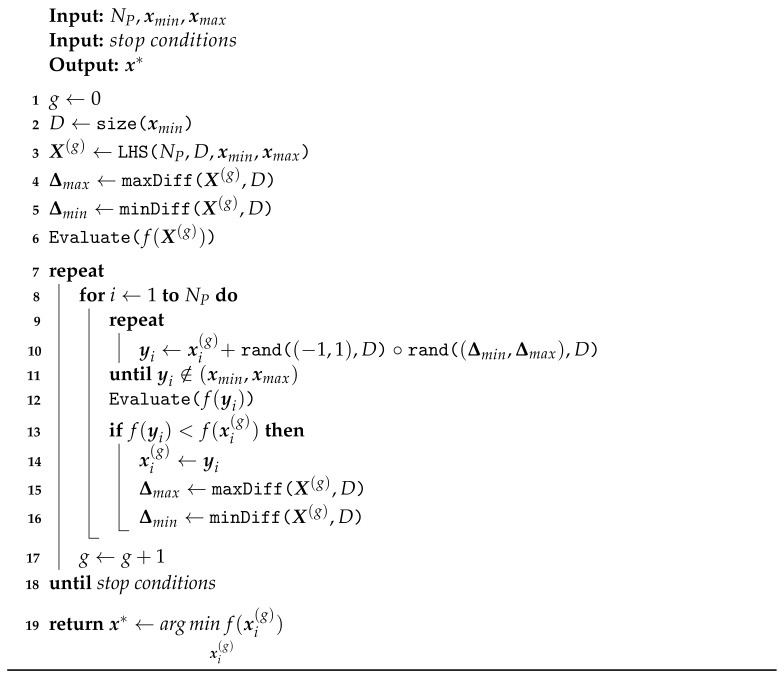



The algorithm uses a single population X of $N_{P}$ members $x_{i}$ that are sampled in the $g=0^{th}$ generation within the bounds ${\left[x_{min},x_{max}\right]}^{D}$ using Latin hyper-cube sampling (LHS) [54] so that a pseudo-random initialisation is achieved. The LHS guarantees a diverse but search-space-representative population considering the population size and problem dimensionality *D*. A random initialisation does not produce optimal coverage and may result in crowds of similar solutions that will attract new solutions and quickly bring optimisation to a halt. The search strategy is built on the DE/current/1/variant [48,55] as a mutation-only strategy described in Equation (17) as

(17) $$y_{i}=x_{i}^{\left(g\right)}+{\left[\mathrm{sign}\left(U^{D}\left(-1,1\right)\right)\circ U^{D}\left(\Delta_{min}^{\left(g\right)},\Delta_{max}^{\left(g\right)}\right)\right]}_{i}\;,$$

where $y_{i}$ is the generated trial solution, ∘ is the Hadamard product, U(·) is a uniformly distributed random value, $i\in \{1,\cdots ,N_{P}\}$, and $\Delta_{max}$ and $\Delta_{min}$ are maximum and minimum vector differences in the current generation $X^{\left(g\right)}$, defined as

(18) $$\Delta_{max}^{\left(g\right)}=\max\left(X_{j}^{\left(g\right)}\right)-\min\left(X_{j}^{\left(g\right)}\right),\;j\in \left\{1,\cdots ,D\right\},$$

and

(19) $$\Delta_{min}^{\left(g\right)}=\min\left(X_{j}^{\left(g\right)}-\min\left(X_{j}^{\left(g\right)}\right)\right),\;X_{j}^{\left(g\right)}\notin \min\left(X_{j}^{\left(g\right)}\right).$$

The current target for replacement $x_{i}$ is chosen as the base vector for a new trial once in every generation, ensuring no selection bias is present [56]. Since no crossover is used, the algorithm is rotationally invariant; consequently, no $C_{r}$ parameter is needed. The difference vector range in each dimension *D* is uniformly sampled from the current extent of difference vectors. In addition, the direction is determined from the signum of uniformly sampled values in the (−1,1) interval. This behaviour mimics the original algorithm but ensures diverse vectors are generated, and no mutation bias is present [56]. The process is equivalent to sampling from an infinite-size population in a bounded search space that can shrink or expand according to the population extent as the search progresses. In line with observations about the generalised scale factor in [55], sampling from a dense population can produce difference vectors equivalent to other mutation operators of varying scale factors, and consequently, the parameter *F* is also not needed. The lower bound on the current range of difference vectors discourages exploitation proportionally to population spread in the search space. It provides simple diversity control to lower the chance of premature convergence by keeping the algorithm in an explorative state for longer. Since the proposed strategy can generate trial vectors with out-of-bound solutions, the mutation operator is repeated on a per-element basis until all constraints are satisfied and the trial can enter the objective function evaluation. The selection process is a local one-on-one competition with the current target $x_{i}$. If better, the trial $y_{i}$ replaces its target in the same generation according to the asynchronous update model and the extent of the search space is updated, so it immediately affects the creation of the next trial vector. The only remaining control parameter that must be provided is the population size $N_{P}$, which can be small. The population members’ role is now to represent the attraction basin and serve as a base vector, while population diversity is provided by sampling difference vectors from the continuous interval.

In the present application of TFD optimisation, the population of $N_{P}=5$ members was experimentally determined as sufficient by observing the convergence rate, population diversity and the objective function variance. In addition, stopping conditions were set conservatively; the objective function variance value-to-reach was set to $1\times 10^{-9}$ and the maximum number of generations to $g_{max}=1\times 10^{5}$. The objective was to test whether reliable convergence of the proposed optimiser could be achieved on different signals using selected performance measures as the objective functions and whether those measures agree with the visual TFD assessment.

Instead of experimenting on different TFDs, we opted for the MTEK. Since it is multiform and tiltable, it represents a general purpose choice second to RGK because it can take on classical and various other kernel topologies using different combinations of its design parameters [30]. The MTEK is defined in the (ν,τ) domain as

(20) $$g_{MTEK}\left(\nu ,\tau \right)=\exp\left\{-\pi {\left[\mu^{2}\left(\frac{\nu }{\nu_{0}},\frac{\tau }{\tau_{0}};\alpha ,r,\beta ,\gamma \right)\right]}^{\lambda }\right\},$$

with

(21) $$\mu \left(\tilde{\nu },\tilde{\tau };\alpha ,r,\beta ,\gamma \right)={\tilde{\nu }}^{2}{\left({\tilde{\tau }}^{2}\right)}^{\alpha }+{\left({\tilde{\nu }}^{2}\right)}^{\alpha }{\tilde{\tau }}^{2}+2r{\left[{\left(\tilde{\nu }\tilde{\tau }\right)}^{\beta }\right]}^{\gamma }.$$

where $\nu_{0}>0$ and $\tau_{0}>0$ are the Doppler and lag scaling constants, and *r* is the tilt parameter usually bounded to the [−1,1] interval. β and γ are coupled powers such that γ=1/β, β∈[1,2], and λ>0 governs the slope of the pass band-to-stop band transition region. α>0 ensures that TFD’s time and frequency marginals are satisfied but since those are of lesser importance in signal processing [1], we set α=0. The boundary constraints on the other MTEK parameters, as used in the tests, are given in Table 1.

Besides the proposed performance measure, optimisations were also performed using the volume normalised Rényi entropy with order α=3 calculated as [1]

(22) $$RE3DV=-\frac{1}{2}\log_{2}\sum_{n}\sum_{k}{\left(\frac{\rho_{z}\left[n,k\right]}{\sum_{n}\sum_{k}\left|\rho_{z}\left[n,k\right]\right|}\right)}^{3},$$

and Stanković’s ECM calculated according to Equation (23) as

(23) $$ECM=\frac{1}{NM}{\left(\sum_{n}\sum_{k}{\left|\frac{\rho_{z}\left[n,k\right]}{\sum_{n}\sum_{k}\rho_{z}\left[n,k\right]}\right|}^{\frac{1}{2}}\right)}^{2}.$$

Equations (22) and (23) represent two of the more commonly used application-agnostic measures when objectively evaluating the performance of a (t,f) distribution and optimising the representation. Commonly used measures are rooted in probability theory; hence (t,f) distributions are viewed as two-dimensional probability density functions satisfying the unit sum constraint [1].

The Rényi entropy is a generalisation of Shannon’s; the idea is that a smaller entropy value is associated with a better-performing distribution or a smaller number of signal components [1]. Typically, the lowest entropy order used is three since order two produces a zero entropy value for a normalised distribution. In contrast, order one is the Shannon entropy, which cannot be applied to distributions containing negative values. For odd-ordered entropy, oscillatory cross-terms in a QTFD cancel out, so different normalisations were introduced to capture their influence: prevalently, the signal energy- and volume-normalised versions. Given that the optimisation guided by the signal energy normalised the Rényi entropy results in the Wigner–Ville distribution as optimal, we used volume normalisation as in Equation (22) to capture the influence of cross-terms and distinguish better-performing distributions past the Wigner–Ville.

Stanković’s ECM, as in Equation (23), is another prevalent measure based on lower-order *ℓ* norms used in signal sparsity measurements [1]. Stanković proposed this measure as an alternative to kurtosis-type measures sensitive to varying powers of signal components. The idea is that better-concentrated distributions have shorter time and frequency support regions, and by measuring their extents, the energy concentration can be estimated, and better performance is associated with lower values of the measure. The lower-order form, as in Equation (23) with power term two, is commonly preferred since it is less sensitive to small TFD values.

## 3. Results

As test signals for the proposed algorithm, we used two synthetic and one real-life multi-component signal. For every considered signal and selected performance measure, 30 optimisation runs were conducted to assess the convergence of the optimiser and the quality of the produced TFDs. The algorithm was implemented in MATLAB 2020b, and signals were processed using Intel Haswell Core i7–4790K desktop processor with 16 GB of RAM. Most of the computational burden corresponds to calculating a time-frequency distribution via fast Fourier transform (FFT). Hence, the overall algorithm has O((NM)log(NM)) time complexity, whereas the selected measures are O(NM). However, differences in the reported average count of generations needed to reach the defined stopping condition are consequences of difficult local optima and plateaux in the objective function landscape, a function of a selected measure and kernel parameters. The results are presented in the sequel.

### 3.1. Linear FM Laws

In order to evaluate the algorithm’s behaviour when there are only linear frequency modulations present, we considered a synthetic signal with two crossed linear FM components of unit amplitude and equal time support. The first component starts at 0.1 Hz and linearly increases with time to 0.4 Hz, while the second component starts at 0.4 Hz and decreases toward 0.1 Hz. The signal was obtained as a discrete sequence of N=128 samples at a 1 Hz sampling rate. Its analytic form is given as

(24) $$z_{I}\left[n\right]=z_{1}\left[n\right]+z_{2}\left[n\right],$$

where the components are

(25) $$\begin{array}{cc} z_{1}\left[n\right] & =\exp\left(j2\pi \left(0.1\left(n-63\right)+\frac{3}{2540}\left(n^{2}-63^{2}\right)\right)\right),\;0\leq n\leq 127, \end{array}$$

(26) $$\begin{array}{cc} z_{2}\left[n\right] & =\exp\left(j2\pi \left(0.4\left(n-63\right)-\frac{3}{2540}\left(n^{2}-63^{2}\right)\right)\right),\;0\leq n\leq 127. \end{array}$$

**Figure 2:**
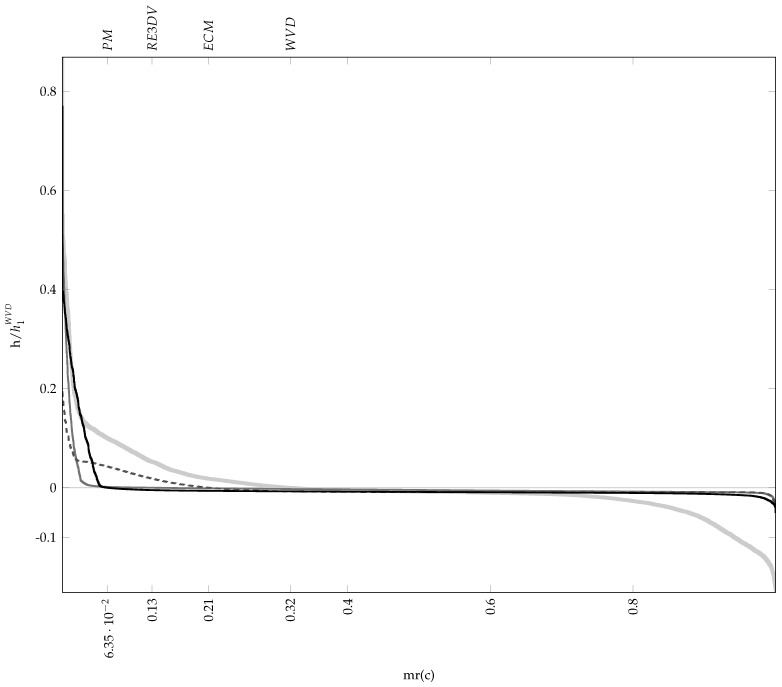
The material ratio curves for the best MTEK TFDs of the two–component LFM signal $z_{I}\left[n\right]$ as in Equation (24). The curves correspond to the TFDs optimised according to the following: RE3DV (grey), ECM (dashed grey) and PM (black). Thick grey is the WVD given for reference. The respective zero–level material ratio mr(0) related to the $p_{3}$ metric is marked on the horizontal axis. A short, steep transition, followed by an extended, nearly flat region, is characteristic of a better–performing distribution.

**Figure 3:**
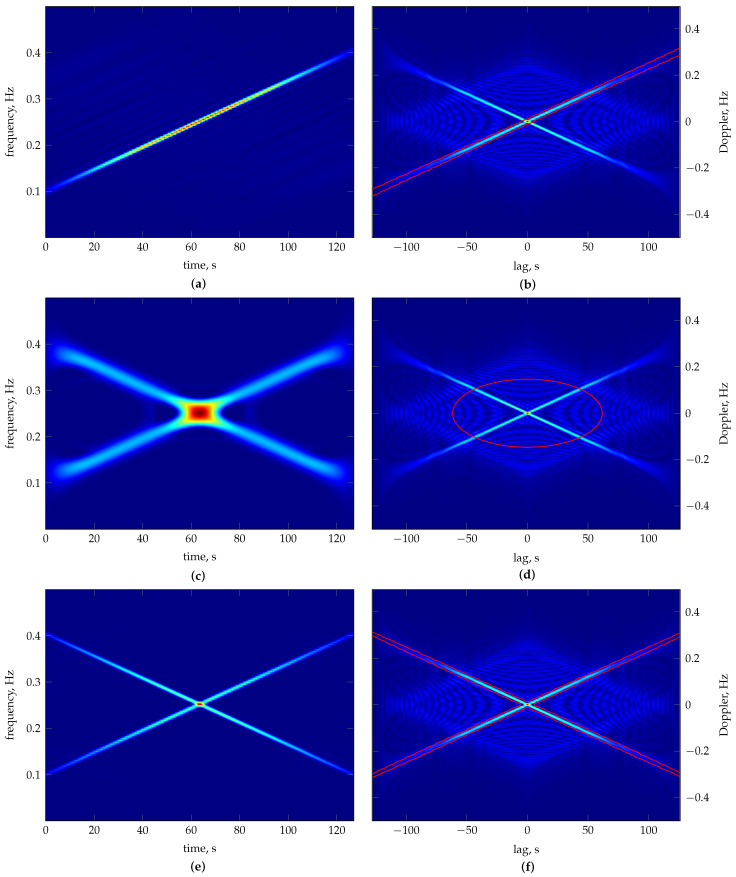
Heat maps of the best MTEK TFDs of the two–component LFM signal $z_{I}\left[n\right]$ as in Equation (24), optimised according to the following: (**a**) RE3DV, (**c**) ECM and (**e**) PM; (**b**,**d**,**f**) show the respective kernel iso–contour at 0.1% maximum kernel level (red line), overlaying the signal ambiguity function magnitude plot. Dark blue is zero level. 1 Hz sampling rate. Individual best kernel parameters are given in Table 3. In (**a**), component $z_{2}\left[n\right]$ (Equation (26)) is completely smeared, whereas $z_{1}\left[n\right]$ (Equation (25)) is highly concentrated. In (**c**), $z_{1}\left[n\right]$ and $z_{2}\left[n\right]$ are visible but lack concentration. In (**e**), both components are well–resolved and highly concentrated.

The average results from 30 repeated runs are reported in Table 2, while Table 3 presents the worst and best results according to and evaluated by each considered measure. Figure 2 shows the material ratio curves of the TFDs generated according to each measure’s best solution. The resulting TFDs are presented in Figure 3, along with the 0.1 per cent MTEK iso-contour overlaying the signal ambiguity function. The number of frequency bins used in generating TFDs is M=128. The optimiser’s behaviour shows consistency for each measure, as evident by comparing the average fitness in Table 2 to the respective measure’s best value in Table 3, verifying that it can converge reliably.

In the parameter space, the ECM results show the smallest variance and the measure took the least amount of generations to reach the stopping conditions. On the other hand, the parameters according to the RE3DV exhibit more significant variance, which is not reflected in the objective function space. Different TFDs result from different parameter values but have similar fitness values, and there are also inconsistencies in the perceived TFD quality and reported numerical values. The RE3DV evaluates PM results as better than ECMs, but among ECMs, it labels the worst as better. According to the ECM, PMs are worse than ECMs, although a visual inspection shows a significantly lower concentration of the ECM result in Figure 3c. According to the RE3DV, the best PMs are second to the RE3DV performance. However, a visual inspection of Figure 3a shows that one component is missing in the RE3DV result, which is why the PM labels ECMs and the WVD as better than RE3DVs. In the considered example, the PM shows greater consistency than the other two measures when the agreement among the perceived performances of results in Figure 3 and numerical indicators in Table 3 are considered.

### 3.2. Mixed FM Laws

As, in practice, signals can have various frequency modulations, we considered a mix of two quadratic components and one linear FM component in the second test. The first component has a quadratic FM law that passes through (t,f) points: (0 s, 0.05 Hz), (63 s, 0.1225 Hz) and (127 s, 0.05 Hz). The second component’s frequency linearly increases with time from 0.2175 to 0.28 Hz, and the third component is, again, a quadratic FM wherein the frequency passes through (t,f) points: (0 s, 0.45 Hz), (63 s, 0.37 Hz) and (127 s, 0.45 Hz). All components are of unit amplitude and the same time support. The signal was obtained as a discrete sequence of N=128 samples at a 1 Hz sampling rate. The analytic form is

(27) $$z_{II}\left[n\right]=z_{1}\left[n\right]+z_{2}\left[n\right]+z_{3}\left[n\right],$$

where the components are

(28) $$\begin{array}{cc} z_{1}\left[n\right] & =\exp\left(j2\pi \left(\frac{1}{20}n+\frac{3683}{3225600}n^{2}-\frac{29}{4838400}n^{3}\right)\right),\;0\leq n\leq 127, \end{array}$$

(29) $$\begin{array}{cc} z_{2}\left[n\right] & =\exp\left(j2\pi \left(\frac{87}{400}\left(n-63\right)+\frac{1}{4064}\left(n^{2}-63^{2}\right)\right)\right),\;0\leq n\leq 127, \end{array}$$

(30) $$\begin{array}{cc} z_{3}\left[n\right] & =\exp\left(j2\pi \left(\frac{9}{20}n-\frac{127}{100800}n^{2}+\frac{1}{151200}n^{3}\right)\right),\;0\leq n\leq 127. \end{array}$$

**Figure 4:**
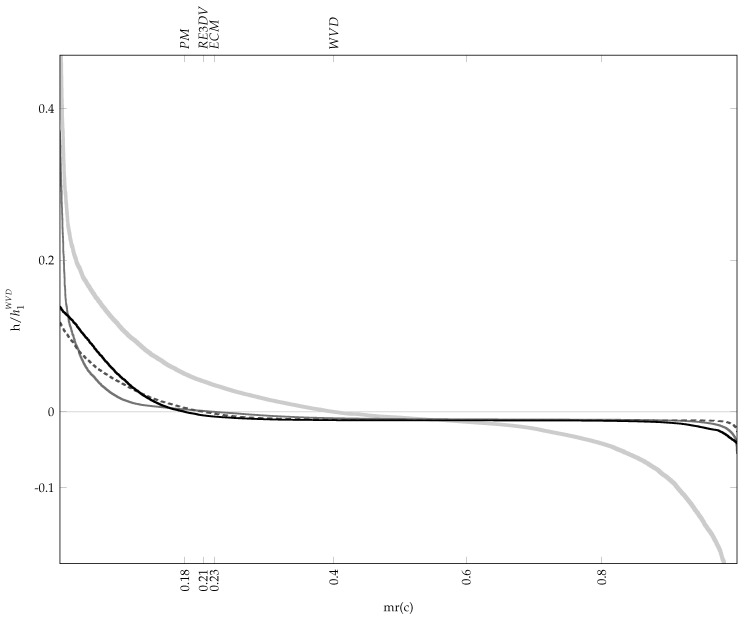
The material ratio curves for the best MTEK TFDs of the three–component mixed FM laws signal $z_{II}\left[n\right]$ as in Equation (27). The curves correspond to the TFDs optimised according to the following: RE3DV (grey), ECM (dashed grey) and PM (black). Thick grey is the WVD given for reference. The respective zero–level material ratio mr(0) related to the $p_{3}$ metric is marked on the horizontal axis.

**Figure 5:**
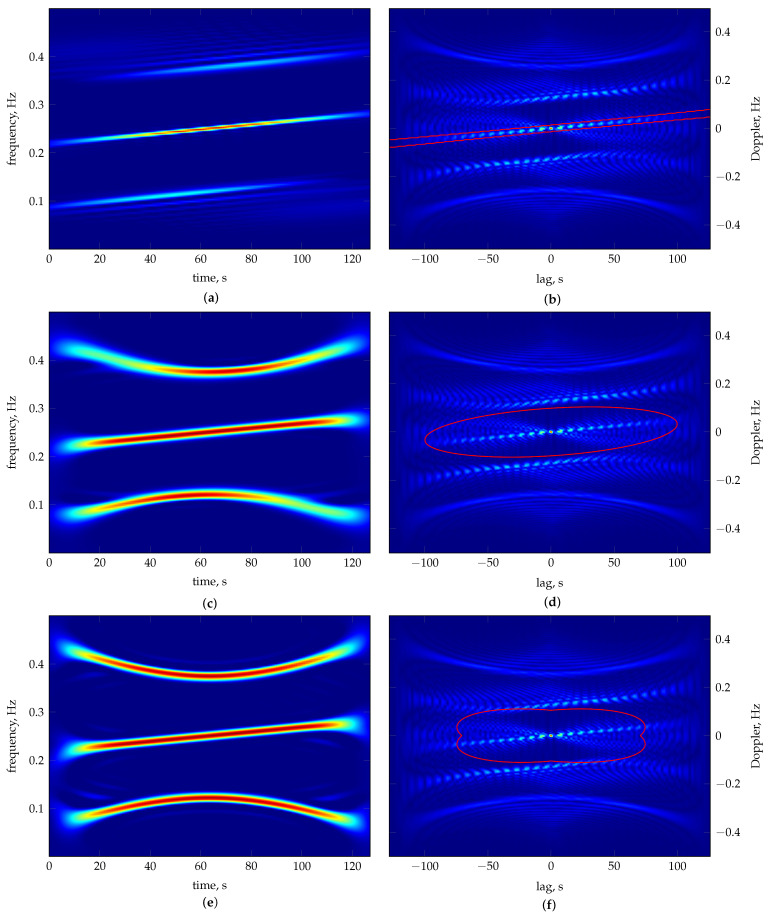
Heat maps of the best MTEK TFDs of the three–component mixed FM laws signal $z_{II}\left[n\right]$ as in Equation (27), optimised according to the following: (**a**) RE3DV, (**c**) ECM and (**e**) PM; (**b**,**d**,**f**) show the respective kernel iso–contour at 0.1% maximum kernel level (red line), overlaying the signal ambiguity function magnitude plot. Dark blue is zero level. 1 Hz sampling rate. Individual best kernel parameters are given in Table 5. In (**a**), linear FM component $z_{2}\left[n\right]$ (Equation (29)) is highly concentrated, but both quadratic FM components $z_{1}\left[n\right]$ (Equation (28)) and $z_{3}\left[n\right]$ (Equation (30)) are significantly smeared and deformed. In (**c**), components are maintained, but there is a loss of concentration for $z_{1}\left[n\right]$ and $z_{3}\left[n\right]$. In (**e**), all components are equally concentrated within their respective (t,f) support regions and are of the correct shape.

The average results from 30 repeated runs are reported in Table 4, and Table 5 shows the worst and best results according to and evaluated by each considered measure. Figure 4 shows the material ratio curves of the TFDs generated according to each measure’s best solution. The resulting TFDs are presented in Figure 5, along with the 0.1 per cent MTEK iso-contour overlaying the signal ambiguity function. The number of frequency bins used in generating TFDs is M=128. As in the case of the crossed LFMs, the optimiser shows reliable convergence for each measure, confirmed by how close the average fitness values in Table 4 are to the respective measure’s best value in Table 5, which indicates it can deal with signals of mixed FM laws.

When the parameter space is considered in Table 5, the values obtained under each measure show low variance, reflected in the objective function space. Hence, no ambiguity is present—similar parameters produce similar results. However, as in the previous example, mutual evaluations show inconsistencies and are not aligned with the visual evaluation. As evident in Figure 5a, under RE3DV-guided optimisation, the MTEK emphasises linear FM segments resulting in a highly concentrated LFM component. However, two quadratic FMs are distorted, and cross-terms are amplified. Similarly, but to a lesser extent, this can also be observed in Figure 5c, which shows the resulting TFD of the ECM-guided optimisation. Here, the quadratic FMs have their shape preserved, but there is a significant loss in concentration toward the components’ edge as if an amplitude modulation is present. On the other hand, the PM result in Figure 5e shows no such effects. All components are equally well concentrated, and their shapes are preserved from start to finish, corresponding to the ground truth. Nevertheless, RE3DV and ECM label PMs as second after their best results, whereas the PM values consistently reflect the perceived performance of TFDs in Figure 5.

### 3.3. Real-Life Signal

For a real-life test case, we used an echolocation signal emitted by the big brown bat (Eptesicus Fuscus) since it is commonly used as an example in the performance assessment of time-frequency processing methods. The signal has four nearly hyperbolic FM components with significant variations in the components’ amplitude and duration. The signal length is 2.5 ms in N=400 samples obtained at a 142.857 kHz sampling rate. The number of frequency bins used in generating TFDs is M=512.

**Figure 6:**
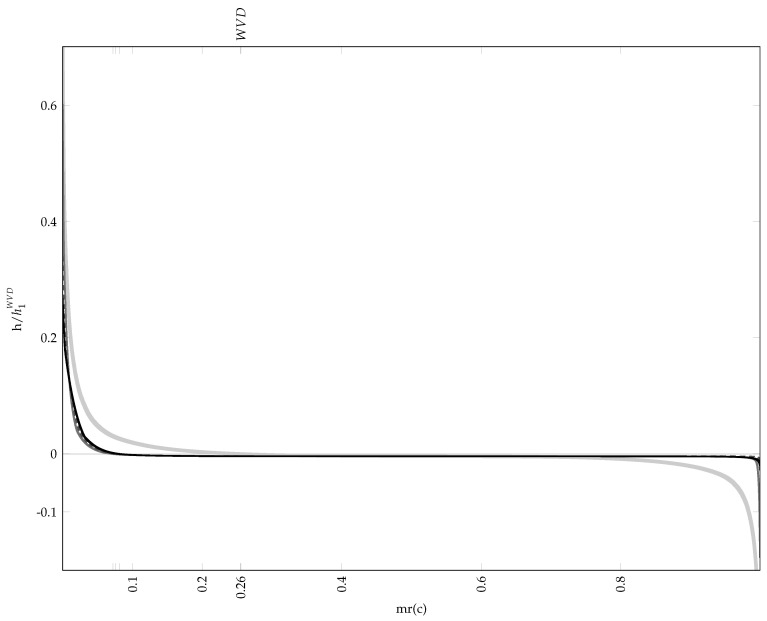
The material ratio curves for the echolocation signal as emitted by the big brown bat. The curves correspond to the TFDs optimised according to the following: RE3DV (grey), ECM (dashed grey) and PM (black). Thick grey is the WVD given for reference. Zero–level material ratio mr(0) related to the $p_{3}$ metric is marked on the horizontal axis only for the WVD. The near coalescence of the individual curves reflects the overall similarities of the resulting TFDs, but the far–right edge reveals significant cross–terms in the RE3DV case.

**Figure 7:**
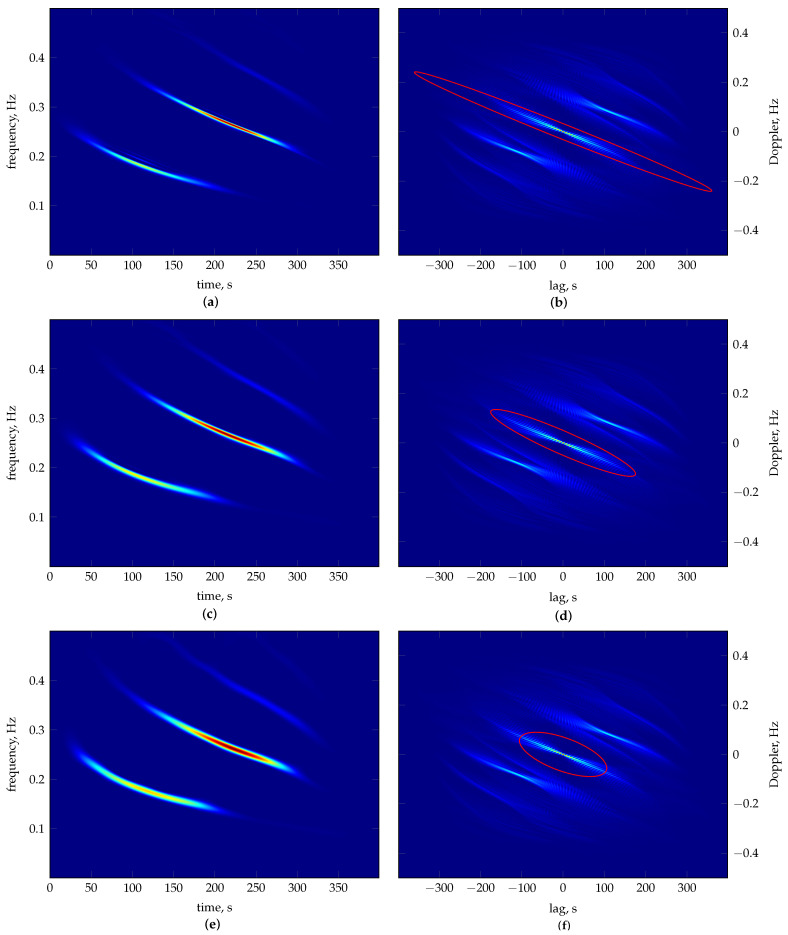
Heat maps of the best MTEK TFDs of the echolocation signal as emitted by the big brown bat, optimised according to the following: (**a**) RE3DV, (**c**) ECM and (**e**) PM; (**b**,**d**,**f**) show the respective kernel iso–contour at 0.1% maximum kernel level (red line), overlaying the signal ambiguity function magnitude plot. Dark blue is zero level. The signal is 2.5 ms long and sampled at 142.857 kHz. The axes are in normalised units. Individual best kernel parameters are given in Table 7. In (**a**), weaker and non–linear segments of the components are smeared, and there are cross–terms near the high–power segments. The components’ shapes are better discernible in (**c**). However, there is some loss of concentration at the weaker segments along with the noticeable cross–term near the high–power segment of the lowest component. In (**e**), the components are equally concentrated in all segments regardless of varying components’ amplitudes, and there are no observable cross–terms.

The average results from 30 repeated runs are shown in Table 6, while Table 7 shows the worst and best solutions according to and evaluated by each considered measure. Figure 6 shows the material ratio curves of the TFDs generated according to each measure’s best solution. The resulting TFDs are presented in Figure 7, along with the 0.1 per cent MTEK iso-contour overlaying the signal ambiguity function. As in the previous examples, the average fitness values in Table 6 correspond well with the respective measure’s best value in Table 7, confirming, on average, the consistent behaviour of the optimiser.

The parameter space in the case of the RE3DV and ECM shows low variance, which is also reflected in the objective function space with no ambiguity in results. The worst of the PMs shows some parameter deviation, but this is appropriately differentiated in the objective function space, so there is also no ambiguity. The RE3DV and ECM label PM results as the worst or second after their best, respectively, but a closer visual inspection of TFDs in Figure 7 reveals a bias toward linear segments in the case of the RE3DV result in Figure 7a and to a lesser extent in the ECM result in Figure 7c. Consequently, subtleties in components’ shapes are diminished, cross-terms appear near strong components, and weaker ones are smeared. In contrast, the PM result in Figure 7e shows equally well-concentrated components regardless of amplitude and duration. Per the reported PM value, it is less distorted than the other two results. The worst PM result (not shown) has a four-quadrant symmetric MTEK topology similar to the one in Figure 7f that passes some cross-terms between the two weakest components, which the PM appropriately captures as lower performance.

## 4. Discussion

The proposed optimiser as a minimalistic differential evolution variant performed reliably in the considered examples regardless of the signal and performance measure as the objective function, signifying how algorithmic simplicity and a small population are not performance-limiting. Since it requires no additional control parameters, it represents a genuine black-box optimiser that, coupled with a reliable TFD performance measure and an adaptable kernel, could be integrated as a service in unsupervised classifiers based on (t,f) signal processing. When the presented results are considered, it is evident how commonly used measures capture only some TFD surface complexities per their definitions, leading to inconsistent behaviour and possible deformations in the resulting TFDs, even in the noiseless case. However, the fact is that no single parameter alone can fully describe all of the surface complexities as recognised in the field of surface metrology. In the proposed measure, four indicators derived from the Abbot–Firestone curve, similar to volume parameters in surface metrology, are aggregated into a single metric. When applied in the considered examples, consistent behaviour was demonstrated by correctly capturing and numerically reflecting situations such as a missing component, increased cross-terms and low concentration. As no additional parameters are required, the proposed measure is a good candidate for a black-box TFD optimiser. However, an additional analysis should be performed for noisy signals and other combinations of components with different frequency and amplitude modulations and varying time support. Furthermore, the MTEK could be replaced by the RGK, which would be an even more significant challenge given the number of kernel parameters. However, optimised correctly, a fully adaptable kernel topology could provide even better-performing time-frequency distributions.

## Figures and Tables

**Table 1:** MTEK boundary constraints as used in the tests. ϵ is the floating-point relative accuracy; here $\epsilon =2^{-52}$. *M* is the number of frequency samples in $\rho_{z}\left[n,k\right]$.

	$\tau_{0}$	$\nu_{0}$	*r*	β	λ
lower	ϵ	ϵ	−2	1	ϵ
upper	*M*	0.5	*M*	2	*M*

**Table 2:** Average results from 30 optimisation runs of the MTEK of $z_{I}\left[n\right]$ according to the considered performance measures.

Generations				MTEK Parameters				
	**Count**	**Time, s**	**Fitness**	$\tau_{0}$	$\nu_{0}$	***r***	β	λ
RE3DV	13,072	127.884	9.27936	4.15204	0.00992	0.46679	1.27949	2.97720
ECM	272	2.221	0.31175	41.50994	0.10006	0.00040	1.24705	0.50000
PM	6225	51.121	0.10211	4.24119	0.01001	−0.99895	1.71353	1.77717

**Table 3:** Performance and kernel parameters of the worst (w) and best (b) resulting MTEK TFDs of $z_{I}\left[n\right]$, optimised and evaluated according to the considered measures. Values for the WVD are given as references. Lower values indicate better performance.

		Performance Measure			MTEK Parameters				
		**RE3DV**	**ECM**	**PM**	$\tau_{0}$	$\nu_{0}$	***r***	β	λ
WVD		11.84341	2.61445	0.23384					
RE3DV	w	9.38104	0.56060	0.26601	7.86578	0.01863	1.00009	1	0.70808
	b	9.27284	0.55657	0.26032	3.98921	0.00954	−1.00029	1	3.07726
ECM	w	10.98149	0.31211	0.21579	41.32846	0.10412	0.01290	1	0.49995
	b	10.99695	0.31159	0.21064	41.60175	0.09905	0.00022	1	0.50000
PM	w	10.29756	0.42100	0.11908	15.71503	0.03433	−0.97847	2	0.64874
	b	9.57260	0.46327	0.10147	3.86339	0.00921	−0.99964	2	1.84672

**Table 4:** Average results from 30 optimisation runs of the MTEK of $z_{II}\left[n\right]$ according to the considered performance measures.

Generations				MTEK Parameters				
	**Count**	**Time, s**	**Fitness**	$\tau_{0}$	$\nu_{0}$	***r***	β	λ
RE3DV	892	8.632	10.47281	21.06995	0.01039	−1.00682	1.22737	1.26482
ECM	251	2.055	0.33449	64.06422	0.06596	−0.31185	1.23792	0.50053
PM	587	4.978	0.16258	56.49719	0.08393	−0.31813	1.73052	0.88273

**Table 5:** Performance and kernel parameters of the worst (w) and best (b) resulting MTEK TFDs of $z_{II}\left[n\right]$, optimised and evaluated according to the considered measures. Values for the WVD are given as references. Lower values indicate better performance.

		Performance Measure			MTEK Parameters				
		**RE3DV**	**ECM**	**PM**	$\tau_{0}$	$\nu_{0}$	***r***	β	λ
WVD		13.04230	3.45997	0.28887					
RE3DV	w	10.47305	0.51045	0.25009	20.64911	0.01021	−1.00636	1	1.32605
	b	10.47276	0.50665	0.25085	21.05173	0.01038	−1.00678	1	1.25676
ECM	w	11.30909	0.33462	0.19700	63.08161	0.06636	−0.30376	1	0.50096
	b	11.29382	0.33445	0.19694	63.96842	0.06625	−0.31692	1	0.50048
PM	w	11.23558	0.44144	0.16283	54.24889	0.07527	−0.41876	2	0.86563
	b	11.24643	0.45091	0.16249	56.49529	0.08397	−0.31773	2	0.88104

**Table 6:** Average results from 30 optimisation runs of the MTEK of the big brown bat echolocation signal according to the considered performance measures.

Generations				MTEK Parameters				
	**Count**	**Time, s**	**Fitness**	$\tau_{0}$	$\nu_{0}$	***r***	β	λ
RE3DV	1197	152.673	11.94109	36.45616	0.02451	0.99085	1.30626	0.69594
ECM	426	43.227	0.14310	44.84364	0.03482	0.92601	1.26103	0.51333
PM	399	41.902	0.08809	59.67790	0.05091	0.49992	1.33746	0.63507

**Table 7:** Performance and kernel parameters of the worst (w) and best (b) resulting MTEK TFDs of the big brown bat echolocation signal, optimised and evaluated according to the selected measures. Values for the WVD are given as references. Lower values are better.

		Performance Measure			MTEK Parameters				
		**RE3DV**	**ECM**	**PM**	$\tau_{0}$	$\nu_{0}$	***r***	β	λ
WVD		14.63600	2.06546	0.27846					
RE3DV	w	11.94150	0.18964	0.16707	35.15062	0.02355	0.99194	1	0.68527
	b	11.94102	0.18935	0.16723	36.31514	0.02441	0.99104	1	0.69765
ECM	w	12.37514	0.14327	0.10113	47.50663	0.03698	0.91057	1	0.51526
	b	12.34999	0.14306	0.10384	44.66734	0.03446	0.92767	1	0.51341
PM	w	12.84688	0.22318	0.09408	51.91000	0.04655	−0.69690	2	0.63085
	b	12.70836	0.17187	0.08733	61.20032	0.05197	0.61916	1	0.64434

## Data Availability

The Big brown bat signal data was obtained from Digital Signal Processing group at Rice University and is available at https://www.ece.rice.edu/dsp/software/TFA/RGK/BAT/batsig.bin.Z (accessed on 15 March 2023) with the permission of Curtis Condon, Ken White, and Al Feng of the Beckman Institute of the University of Illinois. The rest of the data presented in this study are available in the supplementary material or can be recreated based on formulae presented in this article.

## Supplementary Materials

The following supporting information can be downloaded at: https://www.mdpi.com/article/10.3390/s1010000/s1.

## Abbreviations

The following abbreviations are used in this manuscript:

(t,f)	Time-frequency
IF	Instantaneous frequency
QTFD	Quadratic time-frequency distribution
SPWVD	Smoothed pseudo-Wigner–Ville distribution
EMBD	Extended modified B distribution
CKD	Compact kernel distribution
STFT	Short time Fourier transform
WVD	Wigner-Ville distribution
TFD	time-frequency distribution
MTEK	Multiform tiltable exponential kernel
RGK	Radially Gaussian kernel
ECM	energy concentration measure
NIR	Normalised instantaneous resolution
LFM	linear frequency modulated
FT	Fourier transform
IAF	instantaneous auto-correlation function
AF	ambiguity function
(ν,τ)	Doppler-lag
MRC	material ratio curve
PM	proposed measure
DE	Differential evolution
LHS	Latin hyper-cube sampling
RE3DV	volume normalised Rényi entropy with order α=3
FFT	Fast Fourier transform
FM	frequency modulated
